# The Evolution and Recent Trends in Acoustic Targeting of Encapsulated Drugs to Solid Tumors: Strategies beyond Sonoporation

**DOI:** 10.3390/pharmaceutics15061705

**Published:** 2023-06-10

**Authors:** Arvin Honari, Shashank R. Sirsi

**Affiliations:** Department of Bioengineering, Erik Johnson School of Engineering, The University of Texas at Dallas, Richardson, TX 75080, USA

**Keywords:** ultrasound, ultrasound targeting, ultrasound drug delivery, sonoporation, microbubbles, acoustic droplet vaporization

## Abstract

Despite recent advancements in ultrasound-mediated drug delivery and the remarkable success observed in pre-clinical studies, no delivery platform utilizing ultrasound contrast agents has yet received FDA approval. The sonoporation effect was a game-changing discovery with a promising future in clinical settings. Various clinical trials are underway to assess sonoporation’s efficacy in treating solid tumors; however, there are disagreements on its applicability to the broader population due to long-term safety issues. In this review, we first discuss how acoustic targeting of drugs gained importance in cancer pharmaceutics. Then, we discuss ultrasound-targeting strategies that have been less explored yet hold a promising future. We aim to shed light on recent innovations in ultrasound-based drug delivery including newer designs of ultrasound-sensitive particles specifically tailored for pharmaceutical usage.

## 1. Introduction

Modern drug-based cancer therapy is rooted in early revolutionary research carried out by physicians in the late 1800s and early 1900s. Drug targeting, a central concept in modern drug delivery, was introduced in the early 1900s by Paul Ehrlich, a German physician and a Nobel Prize winner [1]. He established the basic principles of precision medicine by defining successful drug delivery as a “magic bullet” that removes pathogens without causing deleterious side effects [2,3]. His research primarily focused on the chemical modification of drugs to enhance their binding affinity to specific pathogens, leading to their effective elimination. Ehrlich is known as the father of chemotherapy, as his search for the magic bullet led to the discovery of arsenic acid derivatives used for treating syphilis and cancer [4,5]. Despite his success in developing successful drug compounds, variable dosing and uncontrolled side effects remained ever-present issues. Thus, Ehrlich advised against intravenous injections of his formidable discoveries [2]. Although substantial progress has been made in reducing drug toxicity in healthy tissues since the start of the twentieth century, harmful side effects from systemic delivery remain a major concern today. To address this issue, various approaches have been proposed to create the ideal drug-delivery platform that dispenses the appropriate dosage of a drug in a controlled manner to the necessary tissue for an extended period. Encapsulation of active agents into nanomedicines is rapidly becoming a popular approach to constructing such an ideal system. The focus of this review is on the recent development of ultrasonic methods to more effectively deliver nanomedicine cargo to the target tissue.

## 2. Drug Encapsulation and Nanomedicine

The encapsulation of drugs has been around for well over a century, dating back to when gelatin capsules were developed. Encapsulation is the process of encasing medicines within a protective shell to withstand certain hostile surroundings and prevent premature release of drug content before arriving at the target site [6]. Drug encapsulation led to the development of drug carriers that have evolved tremendously in the past few decades [7]. Drug carriers gradually became a tool to improve the pharmacokinetics of drugs by controlling their water solubility, stability, or delivery rates [8]. While earlier works focused mainly on drug encapsulation for oral intake (typically on the macro scale), newer strategies are developed to improve drug delivery using nanomedicines introduced systemically. 

The remarkable discovery of enhanced permeability and retention (EPR) by Maeda et al. revolutionized the creation of nanomedicines, specifically for targeting tumors. EPR targeting is a method that employs abnormal tumor structure to deliver drugs solely to tumor tissue [9,10]. Although preclinical data were promising, utilizing the EPR effect in clinics was only partially successful. The reasons behind EPR clinical challenges have been studied extensively in the literature. It is mainly related to the exaggeration of the EPR effect in preclinical tumor models and the heterogeneous nature of this effect [11,12]. Relying solely on EPR targeting has proved insufficient in clinical practice [13,14].

While nanomedicine alone did not emerge as a comprehensive remedy for cancer, it has achieved significant milestones. Firstly, it made pharmaceutical scientists look more carefully at cancer biology and better identify and circumvent the biological barriers against drug delivery [15,16,17,18]. Second, nanomedicine paved the way for designing many drug carrier formulations that successfully encapsulate bioactive agents [19,20]. These particles provided a platform for novel targeting and delivery techniques. Many scientists are further manipulating successful drug carriers developed in the nanomedicine era to improve drug delivery to a target tissue. One good example is designing the lipid nanoparticle formulation used in FDA-approved COVID-19 vaccines [21,22,23]. It is important to note that classic nanomedicine is still an active area of research and novel nanoparticle designs are still being investigated or modified to overcome clinical limitations. A few articles have reviewed recent advances in nanomedicine and particle designs [24,25]. However, nanomedicine has branched into major categories as various strategies have been explored to account for EPR targeting limitations. One of these promising strategies is pharmacological and physical co-treatment, which is the focus of this review. In the following, we delve deeper into solid tumor drug delivery after the EPR era. This review thoroughly explores the promising field of pharmacological and physical co-treatment using ultrasound (US) techniques. 

### 2.1. Nanomedicine beyond the EPR Effect

Combination therapies, localized enhancement of vascular permeability, and ligand targeting are some approaches that have been examined in depth to minimize the requirements for the EPR effect. These strategies have been well documented in many studies [26,27,28,29,30,31]. In this review, our focus is on tumor-selective drug release using acoustic methods. This field of research is still in its infancy and has yet to be tested in clinical trials. Drug carriers can be made sensitive to endogenous stimuli specific to tumor tissues or exogenous energies to release their payload locally in tumors. Intrinsic stimuli targeting uses tumor anomalies, such as lower PH, hypoxia, or abnormal enzyme activities, to trigger drug release from carriers [32,33,34]. In contrast, external targeting applies physical stimuli such as heat, magnetism, electricity, or ultrasound locally to tumor tissue and triggers drug release from a stimuli-sensitive carrier [35,36,37]. Heat-sensitive particles, such as temperature-sensitive liposomes (TSLs) Thermodox^®^, are being explored for this purpose [38,39,40] and have reached clinical trials [41]. Thermosensitive drug vehicles have delivered positive results, but they have faced their own set of challenges, which include low heat resolution, the potential of energy dispersion, and difficulties with administering hyperthermia within clinical settings [42]. Although this drug-delivery approach is quite promising, better options for targeting deep tissue have been devised by altering the external stimulus, such as radiofrequency ablation or ultrasound [43]. The advent of active targeting using external stimuli led to the development of a more comprehensive drug delivery strategy called image-guided drug delivery (IGDD). IGDD is an umbrella term for using an imaging modality to guide drug vehicles to the target tissue, trigger the drug release, and visualize the location and the amount of drug release [44]. IGDD could give physicians a platform to rigorously control drug release and adjust the amount of drug unloading by manipulating the external energy source. Heat-triggered drug release cannot constitute an IGDD platform due to the lack of a feedback system. Various imaging modalities were studied to build a real-time IGDD platform to trigger and measure drug release. Among different medical imaging modalities, ultrasound is famous for its cost-effectiveness, high penetration depth, and spatiotemporal precision. 

### 2.2. Improving Delivery by Drug Uncaging

Through investigation, it became clear that one of the key concerns early on in nanomedicine was a lack of drug release from carriers [45]. This posed a problem for liposomal doxorubicin and cisplatin liposomes, two treatments whose efficacy showed only minor improvement compared to their free counterparts [46,47,48]. One of the reasons for this failure was the lack of a release mechanism from the liposomes to make the drugs bioavailable [49,50]. To combat this issue and increase success rates, researchers sought innovative ways to trigger drug unloading upon a stimulus, thus opening up an entirely new field dubbed “drug uncaging” [45,51,52]. Drug uncaging, on-demand drug release, or triggering drug release all refer to the same concept: drug unloading upon excitement with a stimulus. For the rest of this review, we use the term drug uncaging for consistency. Utilizing external energies such as light, heat, or ultrasound to destabilize drug carriers is widely used for drug uncaging [53]. Despite its precision in applications, the role of ultrasound has yet to be fully explored. In this article, we delve into how therapeutic drugs are transported within tumors and evaluate the potential benefits that could arise from using ultrasound as an effective means of uncaging medicine. 

## 3. Drug Transport in Tumor Tissue

### 3.1. Convection and Diffusion

**Figure 1:**
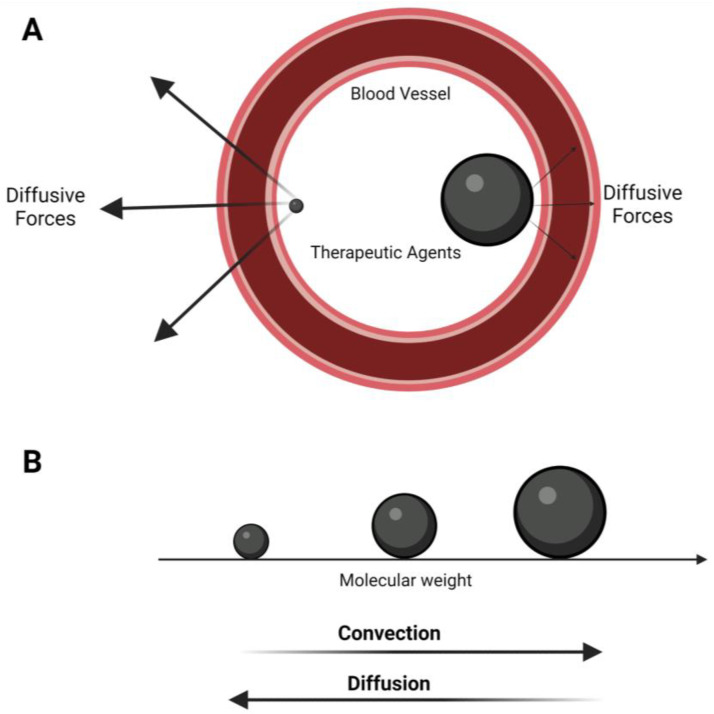
Two major drug transport mechanisms in blood vessels. (**A**) Small therapeutic agents are subjected to stronger diffusive forces than larger ones. (**B**) Molecular weight is directly related to convection and inversely with diffusion.

The two main transport mechanisms for drug delivery to tumors are convection and diffusion [54]. Convection is the process by which fluids move from one area to another due to differences in pressure or temperature. It is often used in drug-delivery systems as it can direct a drug toward its intended target with greater accuracy than diffusion alone. Diffusion, on the other hand, refers to the process by which molecules move from an area of higher chemical potential (partial molar Gibbs free energy) to an area of lower chemical potential [55]. In drug delivery, the concentration gradient of a molecule influences the chemical potential, and typically, active molecules transport from an area of low concentration to an area of high concentration across tissues. This process occurs naturally throughout our bodies but can be enhanced with certain techniques such as ultrasound. Diffusion and convection always coexist in vivo. However, drug and tissue properties dictate which mechanism is the dominant form of transformation. There is a dimensionless number called the Péclet number (Pe), named after a physicist, that predicts which transport mechanism is the primary form. The Péclet number is the ratio of the convective flux to the diffusive flux, in which for Pe > 1, the primary transport mechanism is convection, and for Pe < 1, it is diffusion [55,56]. A drug’s molecular weight (MW) significantly impacts its rate and type of transport [57]. Convective flux is directly related to the MW through the retardation coefficient. Diffusion coefficients are inversely proportional to the hydrodynamic size, demonstrating that smaller molecules have an easier time diffusing. Therefore, larger drugs primarily move through convection, while small drugs typically move by diffusion (Figure 1). As the MW increases, the Pe number also increases, which can affect how drugs interact with cell membranes and ultimately how they are transported. Thus, it is critical to consider the MW when attempting to understand the transport process of a drug [57]. 

### 3.2. Small Molecule Chemotherapy Delivery

Chemotherapeutic molecules have the difficult task of penetrating tumors to reach and destroy the source of cancer. The vascular endothelium is the main barrier that chemotherapeutics must cross to mount an effective attack on tumor tissue. Fickian diffusion is one of the primary means by which drugs diffuse past the vascular endothelium into tumor tissue. Because diffusion is not particularly efficient due to its concentration-gradient-based nature, it requires high drug concentrations in the plasma. Furthermore, the lack of tissue specificity of small molecule chemotherapeutics poses a significant challenge to drug development. Off-target toxicity is a significant concern due to the potential for unwanted side effects that translates into a limitation in dosage in clinical settings. 

### 3.3. Drug Encapsulation: Advantages and Challenges with Nanoparticle Drug Delivery

Encapsulated drugs in nano or micron-size carriers have a Pe number higher than 1 (Pe >> 1), indicating that their transportation is reliant on convective forces and very low in diffusion [58]. However, free drugs usually have Péclet numbers lower than or close to 1 (Pe << 1) and are higher in diffusion. This is one of the primary reasons for the necessity of drug encapsulation. Free forms are drugs rapidly diffuse into the tissue at the injection site before reaching the target. Moreover, most drugs have high lipid solubilities and are inclined to escape from the bloodstream into the tissue, leading to a low circulation half-life. Drug encapsulation drastically improves lipid-soluble drugs’ circulation time by increasing solubility in aqueous solutions [59]. Despite the numerous advantages of drug encapsulation, it can reduce drug bioavailability in target cells and tissues, especially solid tumors. This is because convective forces inside a solid tumor are minimal due to its dense microenvironment, high interstitial pressures, and poor lymphatic drainage, rendering bulk flow negligible [60,61,62]. Consequently, most encapsulated drugs stay on the periphery of the tumor and cannot penetrate deep into the tumor bulk [15]. Solid tumor physiological abnormalities and their effects on drug delivery have been thoroughly characterized in the literature [16,63]. 

Slow convection flow is an established barrier to drug delivery, especially at the center of the tumor. Manual convective flow increase is one solution to this problem and has been explored extensively [64,65]. One of the key ideas to increase the convective flow in the vessels is to use ultrasound waves to open the endothelial cells to enhance the transport of the encapsulated drug (sonoporation effect) [66]. Sound waves can also affect therapeutic molecules. It was found that therapeutic molecules can be dislocated in the direction of ultrasound wave propagation. This effect, coined acoustic streaming, can potentially be used to externally control and guide drug molecules for precise spatial delivery to a target tissue or manually enforce drug penetration in solid tumors [67,68]. 

Another key area of focus for circumventing slow convection is drug uncaging, a promising solution for overcoming large molecule transport obstacles. This approach was initially examined with thermosensitive liposomes [69,70]. Fast drug release creates an intense concentration gradient of small molecule bioavailable drugs, thus changing the dominant mode of transport to diffusive forces. This concept was demonstrated by Manzoor et al., who showed intravascular drug discharge stimulated from temperature-sensitive liposomes in vivo [71], resulting in enhanced uptake levels. The results of the study indicated that intravascular drug uncaging led to a 2-fold increase in penetration depth and increased the duration of contact between drugs and tumor cells by 11–17 fold. This was explained as being due to slower wash-out rates resulting from deeper drug penetration. Other research has likewise proved this idea further, demonstrating a diminished washout rate when employing intravascular drug uncaging [72].

Despite the successful attempt to improve drug internalization, controling thermal drug uncaging can be difficult, especially at greater tissue depths where heat dissipation is a significant problem [42,73]. Additionally, the quick release of drugs can be difficult to manage due to the conflict between the speedy release of particles in vivo and shelf-life stability. An optimal system should be able to instantly unlock its contents yet retain constancy when stored at room temperature. The following section focuses on fresh explorations of using ultrasound to uncage drugs from nanoparticles—a method that resolves some troubles that arise from thermal unpacking. Here, we define ultrasound-mediated drug delivery as any pharmaceutic system that uses ultrasound to enhance the delivery of active molecules to diseased tissue. In this review, we focus on ultrasound-mediated drug delivery that uses ultrasound-sensitive particles, namely microbubbles (MBs) and phase-shift contrast agents (PCAs). These two types of ultrasound-sensitive particles have evolved as ultrasound contrast agents and have been extensively characterized in the literature and used for drug release purposes [74,75,76]. In the following section, we briefly explain ultrasound-sensitive particles, their behavior in the US field, and their application in drug-delivery systems. 

## 4. Ultrasound and Its Contrast Agents

Ultrasound, which consists of inaudibly high-frequency sound waves, has been used for medical imaging for many years. It is primarily used for echocardiography, obstetrics, and abdominal imaging [77,78]. Ultrasound is a safe and cost-effective imaging technique suitable for visualizing the anatomy of deep tissues [79] and blood flow using Doppler imaging [80]. Although ultrasound biomedical research has historically revolved around imaging, the discovery of ultrasound contrast agents has opened new research avenues [81,82,83]. The first ultrasound contrast agent (UCA) was serendipitously discovered in the 1960s when two physicians accidentally imaged bubbles in the bloodstream via a US machine. Gramiak and Shah observed a bright ultrasound signal at the injection site while administering saline to a patient. This group discovered that the intense signal was due to the formation of gas bubbles while shaking the saline solution, suggesting that small gas bubbles serve as ultrasound contrast agents [84]. This discovery subsequently led to the development of surfactant-stabilized microbubbles (MBs) for contrast-enhanced imaging. Ultrasound contrast agents have seen a surge in popularity since the 1990s when the Food and Drug Administration (FDA) first approved their use in contrast-enhanced ultrasound imaging. This approval opened up a wide range of possibilities for drug delivery, enabling targeted and localized treatment of diseases by a technique known as “sonopermeation” [85,86], which is discussed in a subsequent section. In more recent studies, UCAs are being used as efficient drug carriers, enabling researchers to deliver the payload directly to the source of disease in an enhanced and controlled manner [87,88]. Currently, two main types of UCAs, MBs and phase-shift contrast agents (PCAs), are extensively studied for pharmaceutical applications. MBs and UCAs can be loaded with drugs and release their cargo upon ultrasound excitation due to the mechanical cavitation effect. Recent review studies have explored the use of MBs or droplets alone as drug carriers [89,90,91]. 

### Microbubbles and Phase-Shift Contrast Agents

**Figure 2:**
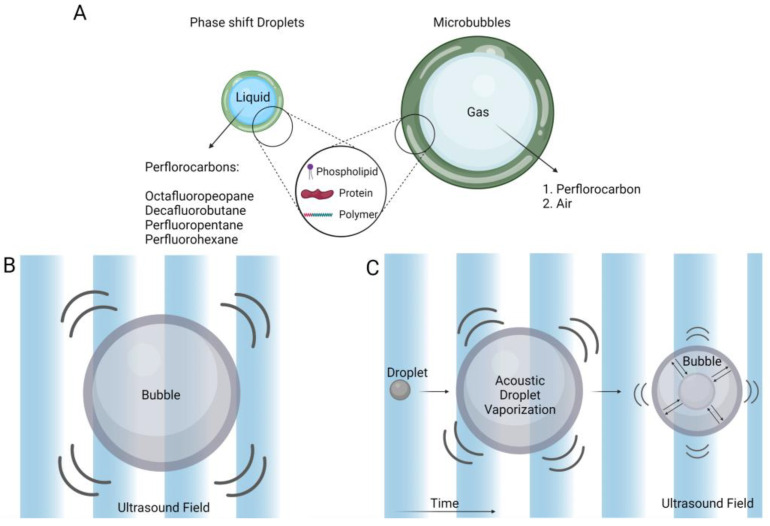
Microbubbles and phase-shift contrast agents’ structure. (**A**) Ultrasound-sensitive agents are encapsulated in a shell for improved stability (**B**) Microbubbles expand and contract in an ultrasound field. (**C**) Phase-shift droplets go through vaporization when excited with ultrasound. They overexpand past their final resting bubble size and then relax at a smaller resting size, where they oscillate as a microbubble.

Microbubbles (MBs) are gas spheres stabilized within a phospholipid, protein, or polymeric shell. MBs go through volumetric oscillation in an ultrasound field (Figure 2). This oscillation displaces the liquid medium surrounding the microbubble and produces a secondary ultrasound field that can disrupt surrounding particles or membranes [92,93]. Thus, MBs are US point scatterers used extensively as contrast agents for US imaging [94]. The response of US contrast agents in an ultrasound field has been thoroughly studied [95,96,97]. Microbubbles are available commercially in several formulations. The first microbubble formulation approved by the FDA was Albunex, consisting of an air bubble entrapped in an albumin shell [98]. Subsequent generations of microbubbles were designed using lipid shells to improve elasticity and fluorocarbon gases for enhanced stability [99]. From several available microbubble formulations widely used in the clinic, one can mention Definity^®^ (Waterloo, ON, USA), Sonovue^®^ (Milan, Italy), and Optison^®^ (St. Louis, MO, USA). 

Phase-shift contrast agents (PCA) are a relatively newer class of particles made of superheated perfluorocarbon liquids. The perfluorocarbons remain in the liquid phase due to the surface tension of the polymer or phospholipid shell and can be vaporized upon US excitation or ambient temperature changes [100]. This phenomenon is called acoustic droplet vaporization (ADV). Upon vaporization, the droplet overexpands (nearly five times) past its final bubble size and then relaxes at a smaller resting size, where it oscillates as an MB [101,102,103] (Figure 2). This vaporization event dislocates the liquid medium and induces high levels of shear force on nearby boundaries or surrounding particles [104,105,106]. Phase-shift droplets have been used for US imaging and US-mediated drug delivery [107]. Following this short introductory section, we delve deeper into ultrasound contrast agents and their most significant accomplishments in drug delivery. We further focus on novel strategies for future endeavors in the field. 

## 5. Ultrasound-Mediated Drug Delivery

To fully understand the benefits of ultrasound agents in drug delivery, it is critical to determine the biological effects of microbubble oscillation and droplet vaporization in the body. Kooiman et al. recently conducted a review to describe and classify the physical and chemical impacts of ultrasound reactive particles on biological tissue [108]. Ultrasound activation of particles can cause various thermal, chemical, and physical changes. However, ultrasound pharmaceutical research is mainly focused on sonoporation (also called ‘sonopermeation’), which has proven highly advantageous.

**Figure 3:**
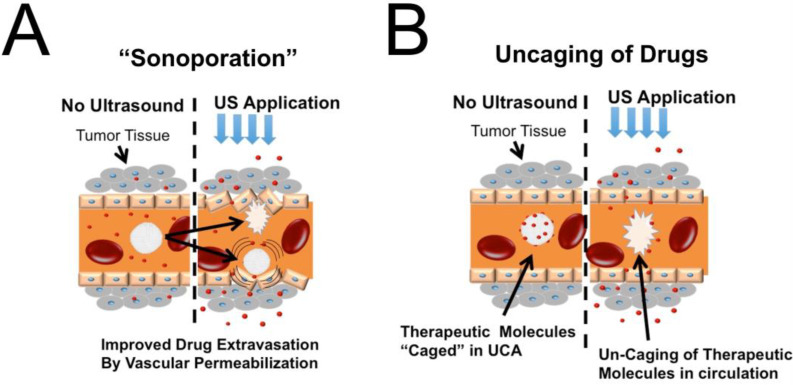
Comparison between drug uncaging and sonoporation strategies (**A**) Sonoporation induces small gaps between endothelial cells to enhance drug convection through diseased tissue. (**B**) Drug uncaging is designed to release drugs rapidly in the bloodstream and create a diffusion gradient for drug transport across the endothelial membrane.

Sonoporation is the most well-known phenomenon in ultrasound drug delivery and consists of “sono” and “poration”, which means inducing gaps using sound energy. Sonoporation in biology means inducing small pores in endothelial cells by ultrasound waves [96] (Figure 3). The sonoporation effect was classically proposed to improve the EPR effect locally in the tumor by generating capillary gaps. Numerous studies have established that MB cavitation or droplet vaporization can improve intracellular drug uptake [106,109,110]. Following the success of sonoporation in preclinical research, studies were conducted to explore the biological mechanisms involved [111,112]. Further studies discovered that sonoporation could induce gaps between confluent cells or the cellular membrane [113], and it became increasingly favored in brain delivery applications due to its potential to open theblood–brain barrier (BBB) [114,115]. Brain sonoporation reached clinical trials and is currently being evaluated as a therapy method for various diseases, such as amyotrophic lateral sclerosis (ALS), Alzheimer’s disease, and glioblastoma (ClinicalTrials.gov Identifiers: NCT04118764, NCT02343991, NCT05615623) [116,117,118]. Sonoporation has also been utilized to enhance the effectiveness of standard chemotherapeutics for treating inoperable pancreatic ductal adenocarcinoma (PDAC) patients in clinical trials (ClinicalTrials.gov Identifier: NCT04821284). Although sonoporation has been successful in preliminary studies and showed positive results in clinical trials, the long-term consequences of this technique are still largely unknown [119]. A few studies have demonstrated the disruption in cell homeostasis as a consequence of sonoporation [120], suggesting that further investigation is essential to completely comprehend the potential adverse effects of sonoporation. A particular study showed adverse effects from opening a specific brain site in rats [121], and a few review papers have warned about ubiquitous clinical BBB opening before studying long-term consequences [122,123]. As Przystupski et al. explored in a recent review, sonoproration provokes various biological responses that are not limited to the cell membrane and include intracellular and intercellular activities. Sonoporation alters the structural and physical integrity of affected cells. It is imperative that it also alters the cell’s function, either temporarily or, at high doses, permanently, an issue that is more critical in the brain. The BBB is one of the most sophisticated and crucial parts of the brain’s evolution history that prevents the brain’s exposure to toxins. Even temporary disruption in the integrity of the BBB might prove detrimental by exposing the most vulnerable part of the human body to pre-existing toxins, such as viruses or bacteria [124,125].

Furthermore, there are arguments in the scientific community that inertial cavitation from microbubbles might enhance metastasis possibilities [126,127]. Concerns about the safety of the sonoporation effect gave rise to an attempt to pursue alternative ultrasound delivery approaches in which the integrity of blood vessels stays intact. Following the tumor vascular normalization idea for cancer therapy, first introduced by Rakesh Jain [128,129], drug delivery to endothelial cells gained popularity. Consequently, ultrasound-mediated drug delivery branched into various techniques to deliver therapeutics without the sonoporation effect. This idea was especially paramount in brain drug delivery as sonoporation is probably most practiced for opening the BBB [115,130,131].

### Materials for Ultrasonic Drug Uncaging

Ultrasonic drug uncaging is only achievable when drugs are embedded in microbubbles/droplets, either on their surface, within the outer shell, or inside the core. The drug loaded onto ultrasound contrast agents (UCAs) rapidly releases after the shell material destabilizes upon ultrasound excitation. The mechanisms by which drug release is triggered depend on ultrasound parameters, and multiple mechanisms have been proposed in the literature, which are not necessarily mutually exclusive. Three major mechanisms are cavitation (which applies high shear forces to the surrounding particles and membranes), thermal ultrasonic effects, and acoustic streaming, resulting in increased frequency and intensity of particle collisions that eventually lead to drug release. The primary mechanism of release is still debated [74]. All the drug vehicles discussed here are designed to utilize these ultrasonic effects to destabilize their structures and release their cargo, in contrast to conventional ultrasonic drug vehicles (such as MBs or PCAs) that directly use ultrasound cavitation as a release mechanism (Figure 3).

One of the primary challenges associated with drug loading solely on MBs or PCAs is the restricted surface area for loading. To enhance payload and target tissue specificity, numerous studies have aimed to develop high-capacity drug-loaded ultrasound contrast agents [132,133,134]. One approach to address the drug capacity issue is to use polymeric microbubbles with thick shells [135]. El-Sherif et al. were the first to develop a polymeric MB formulation [136]. Later, the feasibility of loading high payloads of chemotherapeutics on polymer-based MBs and triggering drug release upon US exposure was demonstrated [137,138,139,140]. More recently, more accessible methods for generating polymeric MBs with potential drug-release applications have been proposed [141,142]. Another approach to improving drug loading on MBs is linking drugs/nanoparticles to the bubbles’ surface (see Chapla et al. [143] for a comprehensive account of drug-loaded microbubble–nanoparticle complexes). The first attempt in this front was conjugating MBs to liposomes, conducted by Kheirolomoom et al. [144] and Lentacker et al. [145]. Other groups showed the feasibility of drug release upon ultrasound excitation using MB–liposome constructs [146,147,148], and they further used it for drug delivery in preclinical tumor models [149,150,151,152,153]. Another novel approach to improving loading capacity is using phase-change droplets with a liquid perfluorocarbon core for loading drugs. Rapoport et al. were the first group to uncage drugs using PCAs after co-loading chemotherapeutics and perfluorocarbons in micelles [154]. This group used drug-loaded (doxorubicin and paclitaxel) polymer nanodroplets to target tumors passively via the EPR effect and triggered drug release upon ultrasound exposure [155,156]. Droplets became a successful alternative for MBs in drug-delivery applications, and many groups showed successful drug delivery to target tissues in preclinical models [157,158,159,160,161]. One of the limitations of PCAs as drug carriers is the limited solubility of drugs in the perfluorocarbon core. To address this issue, our group conjugated droplets to liposomes for the first time to improve droplets’ loading capabilities and demonstrated drug uncaging upon ultrasound application in vitro and in vivo [162].

## 6. Emerging Areas with Ultrasonic Drug Uncaging

Research on the development of drug-loaded ultrasound contrast agents typically serves to augment sonoporation rather than supplant it. However, this area of ultrasonic drug delivery is starting to branch out and develop in new directions. In recent years, a new field has emerged to deliver drugs directly into target sites without harming the vascular tissue, particularly applicable when treating brain diseases where even short-term BBB opening may be considered unsafe or undesired during therapeutic intervention. Airan et al. first introduced and investigated this idea when they uncaged propofol in a rat’s brain [163]. Delivery was carried out by propofol uncaging from polymeric PCAs and was confirmed by monitoring the brain activity via EEG measurements. Intact BBB integrity was confirmed by MRI and histology evaluations. This work was the first attempt to maintain blood vessel integrity after drug uncaging [164]. More recently, Lea-Banks et al. efficiently delivered a barbiturate anesthetic to the mouse brain by vaporizing lipid PCAs with no collateral damage to the BBB, confirmed by MRI and histology [165], and they also showed sub-millimeter precision in the delivery [166].

Microbubbles have also been used for non-invasive drug uncaging. In a recent study, Ozdas et al. demonstrated that low-intensity ultrasound applied to a liposome–microbubbles construct improves localized delivery of an encapsulated GABA_A_ receptor agonist (muscimol) without compromising blood-brain barrier integrity [167]. In another study, Gorick et al. successfully transfected plasmids to endothelial cells in a mouse brain using low-intensity ultrasound and plasmid-loaded MBs [168]. This study showed no BBB opening after US exposure using MRI measurements. Ultrasound-mediated brain drug uncaging without affecting BBB integrity is becoming increasingly popular, especially for gene delivery. 

### 6.1. Incorporating Cavitation Nuclei in Drug-Delivery Vehicles

**Figure 4:**
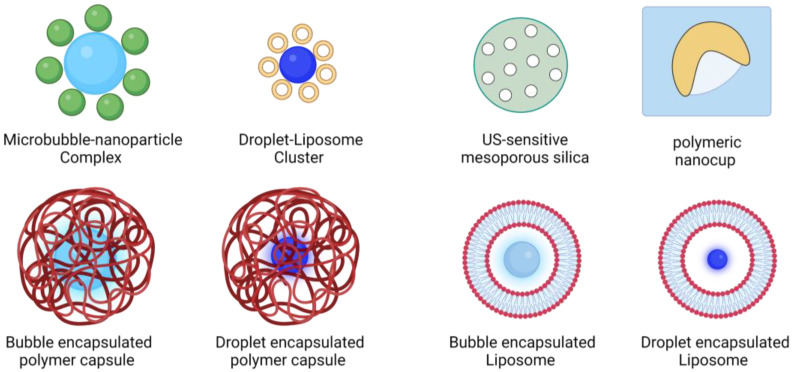
Ultrasound-sensitive particles designed for drug-release applications. Combining ultrasound contrast agents with various drug carriers results in novel ultrasound-sensitive drug vehicles that trigger release upon ultrasound radiation.

As discussed earlier, one of the primary limitations of using conventional US contrast agents as a drug carrier is the low drug capacity. One of the key ideas to address this issue is incorporating ultrasound-sensitive agents inside conventional drug carriers to sensitize them to US waves (Figure 4). 

### 6.2. Microbubble-Nested Drug Carriers

One of the first attempts on this front was encapsulating lipid-shelled microbubbles inside polymeric microcapsules by Wrenn et al. [169]. Although the motivation for this study was primarily to give lipid shell MBs a longer circulation time and shelf stability [170], this group showed some advantages in drug delivery [169]. This group also encapsulated PCAs in microcapsules for potential drug-delivery applications but mainly investigated their imaging applications [171]. Encapsulating bubbles inside liposomes by the same group was the first attempt to nest cavitation nuclei in conventional drug carriers solely for drug-delivery applications. This group reported surrogate drug release in vitro from bubble-nested liposomes upon ultrasound excitation and bubble cavitation [172]. Ibsen et al. also entrapped lipid-shelled microbubbles in liposomes for drug-delivery applications [173]. More recently, Batchelor et al. nested nanobubbles in liposomes to reduce the construct’s size in the submicron range. This group demonstrated drug uncaging upon activation by high-intensity focused ultrasound (HIFU). It is worth noting that this study reports nested nanobubbles to be a mixture of perfluorocarbon gas and droplet [174].

### 6.3. Droplet-Nested Drug Carriers

An alternative approach for sensitizing liposomes to US was nesting PCAs inside them. A few groups encapsulated perfluorocarbon droplets in liposomes and demonstrated successful drug uncaging in vitro [175,176]. The limitation of the abovementioned studies is the lack of in vivo evidence for the feasibility of drug uncaging upon US excitation from ultrasound-sensitive particles nested in drug carriers. Further studies need to address this issue. 

### 6.4. Nesting Cavitation Nuclei in Solid Drug Carriers

Embedding cavitation nuclei in dug vehicles was also pursued in solid drug vehicles. Solid particles can stabilize or nucleate gas within pores or crevices on their surface or mantle to produce acoustically active agents [177]. This phenomenon has been used for drug delivery applications, mainly by entrapping gas bubbles in silica particles (mesoporous silica) and polymeric particles [178]. Mesoporous silica particles have been widely used for drug uncaging [179,180]. Kim et al. did one of the first studies on this front when they delivered ibuprofen from mesoporous silica upon ultrasound activation in vitro [181]. This strategy was further proven helpful in preclinical tumor models [182].

The first approach to embed cavitation nuclei in polymeric particles was developing cup-shaped polymers that can entrap small gas bubbles in their structure. Kwan et al. developed nano-sized polymeric cups to entrap gas bubbles and further demonstrated their ultrasound activity and drug-release capabilities [183]. In another attempt, the same group developed porous PLGA microparticles to trap gas bubbles and potential ultrasound-mediated drug-delivery applications [184]. More recently, our group has developed a facile method to nucleate bubbles inside polymeric microcapsules to produce ultrasound-sensitive drug carriers. We successfully nucleated bubbles in polylactic acid microcapsules by resuspending them in a propylene glycol/glycerol solution. We characterized the formulated particles and demonstrated surrogate drug loading on them. This method can drastically reduce the time and energy of conventional methods [141]. A recent paper by Sabuncu et al. reviewed gas-stabilizing solid particles for pharmaceutic applications [185].

## 7. Materials for Ultrasound-Sensitive Drug Carriers

In the previous sections, we discussed various structures scientists developed to trigger drug release upon ultrasound excitation. In this section, we provide a brief summary of different materials used to create these structures and compare their advantages and disadvantages (Table 1).

### 7.1. Lipids

Phospholipids are commonly used for designing drug carriers and have shown great success in clinical applications, making them one of the primary choices for drug carrier design [186]. In the field of nanomedicine, phospholipids have evolved to be biocompatible and effective in encapsulating active molecules. Phosphatidylcholines (PCs), a class of phospholipids, have been widely used for this purpose [187]. In many of the newer carrier structures discussed in this paper, phospholipids are the predominant material. They are extensively employed in producing complexes of UCAs with nanoparticles [143]. Phospholipid bilayers are also used to encapsulate UCAs, providing stability and increased drug-loading capacity by generating nested UCAs [172,173]. Phospholipids mimic cellular bilayers and possess desirable biocompatibility. Additionally, their elasticity make UCAs from phospholipids respond well to ultrasound signals. However, the drug-loading capacity of phospholipid particles can be limited [188].

### 7.2. Polymers

Polymers are another primary class of materials used in ultrasound-mediated drug delivery. They offer chemical flexibility to drug carriers, allowing modification of their mechanical and physical properties to suit specific biological environments. This chemical flexibility is particularly advantageous in microbubble design, as altering the mechanical properties of the microbubble shell enables control of their response to ultrasound [189]. Polymers also provide the carrier with the unique ability to be biodegradable [190].

Among various polymer formulations, polyesters are attractive polymers extensively used in drug delivery due to their biocompatibility and biodegradability. Poly(lactic-co-glycolic acid) (PLGA), a copolymer of two polyesters, poly(lactic acid) (PLA) and poly(glycolic acid) (PGA), is one of the primary polymers used for generating microbubbles [191]. Another desirable polymer for microbubble production is poly(butyl cyanoacrylate) (PBCA), which is well tolerated by the body and is degradable [139,140,192]. Several groups, including ours, have pursued encapsulating ultrasound contrast agents in a polymeric shell for drug delivery or imaging applications [162,189]. In addition to chemical flexibility and degradability, polymers can offer in vivo stability to a drug carrier. Polymers are rigid structures capable of circulating in the bloodstream for days to months. Due to their entangled structure, they can provide better drug-loading capacity than phospholipids by offering a thicker shell material. However, one drawback of polymers is their reduced reactivity to ultrasound signals due to the lack of elasticity compared to lipids [193]. 

It is worth noting that proteins are also a class of materials used for designing ultrasound-sensitive particles (particularly MBs) in the past but have been a less popular choice for designing UCAs recently [194].

## 8. The Role of Ultrasound Parameters in Drug Vehicle Design

One major consideration for developing ultrasound drug delivery carriers is safety. Ultrasound exposure safety involves two aspects: the bioeffects of ultrasound energy that activates the drug carriers and the bioeffects of cavitation after carrier activation.

The initial designs for acoustic drug delivery with drug carriers utilized the inertial cavitation of bubbles to destabilize the carriers. The first attempt involved co-injecting MBs and drug carriers [195]. Later, MBs were bound to drug carriers to enhance drug release by increasing their proximity [143]. However, this method requires high levels of ultrasound energy, and inertial cavitation itself can be harmful to the tissue due to the strong ultrasound field or shock waves it produces. Concerns about the safety of inertial cavitation led to the use of stable cavitation as a delivery mode. However, the sonoporation effect soon overshadowed this approach, and fewer attempts were made to solely destabilize drug carriers through stable cavitation [196]. Concerns about the sonoporation effect have been extensively discussed in previous sections. The exact ultrasound parameters used in studies utilizing inertial or stable cavitation of MBs to enhance drug release and delivery vary due to different experimental conditions. For more information on ultrasound parameters, please refer to the review paper by Kooiman et al. [197]. 

Another approach to reduce the ultrasound energy is to use perfluorocarbon-based PCAs instead of MBs. The activation threshold of PCAs can be controlled by adjusting the boiling point of the PFC core. Theoretically, one can design a PCA that can be activated at different ultrasound energy levels by altering the chemistry of the fluorocarbon core [100,198]. However, concerns regarding the bioeffects of vaporization effects still exist. 

An alternative approach to reducing the side effects of cavitation is to encapsulate the cavitation effect within a vesicle. This concept led to the nesting of cavitation nuclei within drug carriers. By placing the cavitation nuclei in a vehicle and enclosing the UCAs, the effects of cavitation can be localized and directed solely to the carrier structure, thereby reducing the bioeffects. However, concerns remain regarding the activation threshold of UCAs when embedded in a membrane-bound vesicle. Wrenn et al. observed an increase in cavitation thresholds when MBs were nested within liposomes [199]. This concern can potentially be addressed by utilizing low-boiling-point PCAs with low activation thresholds.

More recently, attention has been given to alternative approaches for ultrasound targeting and delivery that are less invasive and can be used with low-intensity ultrasound. Other targeting modes, such as acoustic streaming or primary acoustic radiation forces, have gained attention as valuable methods for ultrasound-mediated drug delivery. The following section briefly covers recent advances in pharmaceutical uses of acoustic radiation forces.

## 9. Acoustic Radiation Forces in Drug Delivery

Before concluding this paper, we want to briefly define the application of acoustic radiation forces in pharmaceutics and discuss recent advances in this field. Ultrasound can offer a different targeting strategy by spatially controlling and dislocating acoustic-sensitive agents. It is known that MBs can be displaced in a liquid medium using low-pressure acoustic waves due to the absorption of the sound wave momentum. This phenomenon is known as primary acoustic radiation force and is particularly advantageous in drug-delivery applications using low-intensity US waves [200,201]. Radiation forces are one of the less explored concepts in drug delivery despite its promising potential [202]. The concept was first explored in vivo by Dayton et al., where they displaced commercially available microbubbles to the wall of a blood vessel in the mouse cremaster muscle [200]. Later, Lum et al. demonstrated the feasibility of particle deposition on a tube using acoustic radiation [203].

One of the novel methods for ultrasound targeting is utilizing radiation forces to remotely implant sensitive acoustic particles in diseased tissue [142]. In a recent paper, Su et al. successfully implanted multi-cavity PLGA microparticles in a foam-cell spheroid model by ultrasound exposure [204]. The implanted particle can slowly release its cargo to the diseased tissue. Our group is trying a strategy to use acoustic radiation forces to deposit liposomes on endothelial cells, similar to the sonoprinting approach first described by Cock et al. [205]. Liposomes (or any desired drug vehicle) slowly release their cargo to the tumor tissue or the endothelial cell over time. This method uses safe, low-intensity ultrasound waves and the integrity of the endothelial cells is intact as per our hypothesis. We have shown successful lipid deposition onto neuroblastoma tumor cells in vivo. 

## 10. Conclusions

Nanomedicine has provided hope in delivering drugs systemically, but it has only been partially successful clinically. Recent studies have shown that combination therapies, localized enhancement of vascular permeability, ligand targeting, and image-guided drug delivery hold significant promise for improved therapy. Additionally, ultrasound as an option for intravascular drug release has emerged as a cost-effective and precise technique due to its high penetration depth and spatiotemporal precision. The future of drug targeting is exciting, with more technological advancements on the horizon. We can expect to see even more precise therapies and treatments with fewer side effects in the coming years, resulting in better patient health outcomes. 

## Figures and Tables

**Table 1:** Materials used in ultrasound-sensitive drug carrier designs.

Structure	Materials		Pro	Con
UCA nested particles	Nested UCA Shell Material	Phospholipid (DSPC, DPPC)	1. High drug-loading capacity 2. Long circulation time 3. Suitable for encapsulating both hydrophobic and hydrophilic agents	1. Typically micro-size range 2. Limited ultrasound sensitivity
		No shell		
	Encapsulation material	Polymer (PLGA, PLA, PBCA)		
		Phospholipid (DSPC, DPPC, DMPC)		
UCA-Particle complex	UCA	Phospholipid (DSPC, DPPC)	1. Improved ultrasound sensitivity 2. Improved drug release	Limited circulation time compared to polymers
	Drug carrier	Phospholipid (various PCs)		
		Polymer (PLA, PLGA, PEG)		
		Miscellaneous material (metallic particles, proteins, etc.)		
Coned shape particles	Air-entrapped polymer (PLA, PLGA)		1. Biocompatible 2. Long circulation time 3. Biodegradability	1. Large size 2. Limited ultrasound sensitivity
Mesoporous particles	Air-entrapped silica		1. Long circulation time 2. Biocompatible	Limited ultrasound sensitivity
